# In Vitro Biocompatibility of a Novel Semi-Rigid Shell Barrier System: As a New Application for Guided Bone Regeneration

**DOI:** 10.3390/polym14122451

**Published:** 2022-06-16

**Authors:** Rudjit Tunthasen, Prisana Pripatnanont, Jirut Meesane

**Affiliations:** 1 Cranio-Maxillofacial Hard Tissue Engineering Center, Oral and Maxillofacial Surgery Section, Faculty of Dentistry, Prince of Songkla University, Songkhla 90110, Thailand; rudjit0307@gmail.com; 2 Institute of Biomedical Engineering, Faculty of Medicine, Prince of Songkla University, Songkhla 90110, Thailand; jirutmeesane999@yahoo.co.uk

**Keywords:** barrier membrane, biphasic calcium phosphate, guided bone regeneration, polycaprolactone, semi-rigid shell barrier system

## Abstract

This study evaluated the in vitro biocompatibility of a novel, semi-rigid shell barrier system for guided bone regeneration (GBR) based on polycaprolactone and biphasic calcium phosphate membranes and consisting of a semi-rigid shell (SR) and two semi-resorbable barrier membranes, i.e., a buffered (BF) and an airdried (AD) membrane. In vitro biocompatibility, cell cytotoxicity, cell proliferation and differentiation were evaluated with osteoblast (MC3T3-E1) and fibroblast (L929) cells compared to the d-PTFE membrane (Cytoplast^®^, CP). The osteoblasts and fibroblasts were well attached and proliferated on all materials from days 1, 3, and 7 without cell cytotoxicity. All groups showed that osteoblast and fibroblast cell proliferation increased from day 1 to day 14–17 and decreased on day 21. On day 21, the CP membrane presented significantly higher osteoblast cell numbers than the BF membrane and the SR shell (*p* = 0.000). The CP membrane presented a significantly higher amount of fibroblast cells than the other groups (*p* = 0.000). The SR shell and the BF membrane demonstrated higher osteoblast cell differentiation markers including ALP activity, osteocalcin, and mineral secretion than the CP and the AD membrane. The semi-rigid shell barrier system demonstrated good in vitro biocompatibility and supported osteogenic cell proliferation and differentiation better than the d-PTFE membrane.

## 1. Introduction

A barrier membrane is a crucial component in guided bone regeneration (GBR) to exclude the invasion of surrounding soft tissue and allow the migration of pluripotential and osteogenic cells to the defect site to regenerate new bone in bone defects. The rigid structure functions as a cortex and is essential in a non-containing defect for holding grafting materials, maintaining the space, and letting the osteogenic cells form bone. The rigid structures can be either an autogenous bone shell, a bone block or a non-resorbable Ti-reinforced membrane. Although autogenous bone shells or bone blocks are good in terms of strength, ability to integrate with the surrounding bone, and osteogenesis properties, they require second site surgery and the harvesting procedure may lead to donor site morbidity [1,2]. Non-resorbable barrier membranes, for instance, a titanium mesh and a polytetrafluoroethylene (PTFE) membrane, are good in terms of maintaining the bone volume but need later removal, and their rigidity usually causes unwanted membrane exposure [3,4,5,6]. Resorbable collagen membranes easily collapse and cannot maintain the space [3,7]. Polymeric shells or membranes such as polylactic acid (D and L isomers) (PDDL) are resorbable and can maintain the space, but their high rigidity and distortion after use make them unpopular [8,9].

**Figure 1:**
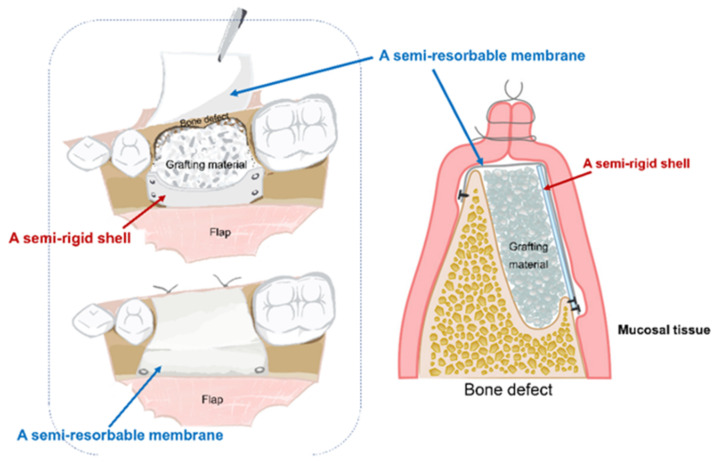
Conceptualization and utilization of a semi-rigid shell barrier system in clinical application.

Based on these limitations, the concept of a novel, semi-rigid shell barrier system was introduced. The system consisted of a semi-rigid shell and a semi-resorbable covering membrane. The semi-rigid shell is fixed to the outer cortex by bone tacks outside the defect to act as a bone wall. Then, bone particles will be filled in the space and are covered with the covering membrane, which functions as a barrier membrane (presented in Figure 1) [10]. The system is designed to have the following properties: biocompatibility, barrier function, enough strength to hold the grafting material and maintain the space, convenience for handling, semi-resorbable, and compatibility with bone [11].

Poly ε-caprolactone (PCL) is a safe polymer for medical use and has been approved by the US Food and Drug Administration, FDA [12]. Biphasic calcium phosphate (BCP), a 2-phase of hydroxyapatite (HA) and bioresorbable β-tricalcium phosphate (β-TCP), is a popular ceramic to combine with polymers to improve the properties of the material, e.g., mechanical properties, reduced acidity from hydrolysis of polymer, and increased osteoconductive properties [12]. PCL and BCP were selected as a base material for a composite, semi-rigid shell barrier system. PCL has good in vitro and in vivo biocompatibility with excellent mechanical properties. PCL completely degrades around 24 months by hydrolysis without producing acid [13,14]. Combining PCL with BCP improves the degradation time from the dissolution of TCP and simultaneously releases Ca^2+^, HPO_4_^2−^, and PO_4_^3−^, which subsequently induces bone formation. As such, the composite material becomes a bioactive material with osteoinductive properties [15,16,17,18,19]. 

A BCP ratio of 30/70 previously showed the best results in osteoblastic proliferation and differentiation [16,19,20]. PCL to BCP at a ratio of 70/30 and 80/20 was comparable in combination with polymers in terms of physical properties and in inducing the differentiation of mesenchymal stem cells to osteoblast cells [19]. In our previous study [10], PCL-BCP (HA/TCP: 30/70) shell and membranes at a ratio of 70:30 demonstrated micropore sizes ranging from 5 to 40 μm, endowing them with a barrier function. They possess hydrophilic properties with a rough surface architecture and suitable mechanical properties with excellent tensile strength as a shell and membrane, depending on the thickness of the material.

This study evaluated in vitro biocompatibility of a novel, semi-rigid shell barrier system consisting of a semi-rigid shell and a semi-resorbable covering membrane, based on a composite material of polycaprolactone and biphasic calcium phosphate (ratio of PCL/BCP: 70:30) for guided bone regeneration (Figure 1).

## 2. Materials and Methods

### 2.1. Materials

- Biphasic Calcium Phosphate (BCP) (HA30: β-TCP:70) with particle size < 75 µm was obtained in the cooperation with the Assistive Technology and Medical Devices Research Center (A-MED), Thailand.
- Poly ε-caprolactone (PCL), ${\bar{M}}_{n}$ 80,000 by GPC was purchased from Sigma-Aldrich, Gillingham, UK.
- A high-density polytetrafluoroethylene (d-PTFE) membrane (Cytoplast^®^ TXT-200, Osteogenics Biomedical, Lubbock, TX, USA), which is a non-resorbable membrane, was selected as a control.

### 2.2. Fabrication of the Semi-Rigid Shell Barrier System

The semi-rigid shell barrier system was fabricated from a composite of poly ε-caprolactone (PCL) and biphasic calcium phosphate (BCP) with the ratio of PCL/BCP: 70:30. The process was based on our previous work [10].

The procedures followed that described in a previous study [10]. Briefly, 70% of PCL and 30% of BCP particles [HA30: β-TCP70] by weight were mixed homogeneously in organic solvent (formic acid: acetic acid = 1:3). The composite solution was then cast in a molding block, to form a sheath structure by the layering casting technique for a shell structure and the covering membranes. The airdried membrane was done by airdrying of the solvent and a buffered membrane was obtained by buffering technique that was immediately changed the acidic condition with buffer. 

The material morphologies were observed by eye and under a scanning electron microscope (SEM). In vitro biocompatibility with fibroblast and osteoblast cells was evaluated. There were four study groups with five samples per group per experimental day for which each test was performed as presented in Table 1.

### 2.3. Material Morphology Evaluation

#### 2.3.1. Gross Observation

The appearance, surface architecture, and flexibility of the material in all groups were observed.

#### 2.3.2. Surface Morphology

A scanning electron microscope (SEM) (Quanta400, FEI, Brno, South Moravian Region, Czech Republic) was used to evaluate the surface morphology of the materials. The samples were cut into small pieces, mounted on a metal stub, and coated with gold.

### 2.4. In Vitro Biocompatibility 

**Figure 2:**
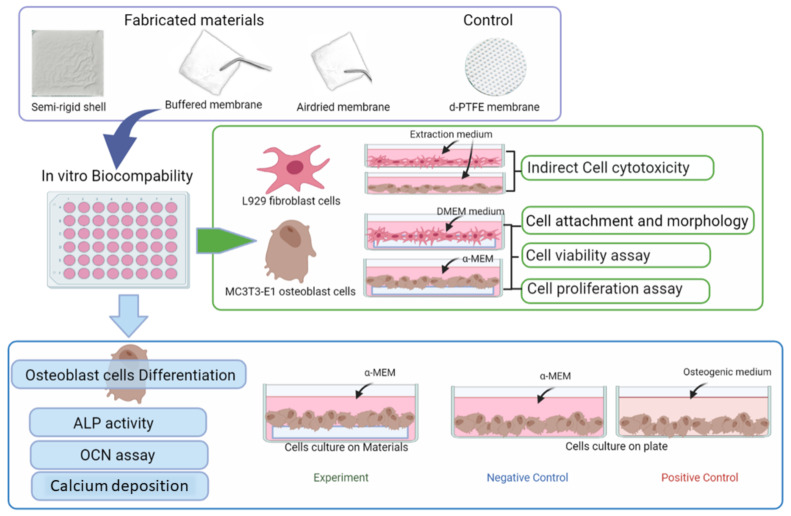
Overview schematic illustration of the in vitro biocompatibility evaluation.

The in vitro biocompatibility test, cell attachment, cell proliferation, and osteogenic differentiation test are summarized in Figure 2. 

#### 2.4.1. Osteoblast and Fibroblast Cell Culture

Fibroblastic cell lines (L929 cell line, ATCC, Manassas, VA, USA) were cultivated in DMEM cultured medium (Dulbecco’s Modified Eagle Medium) with 10% fetal bovine serum (FBS), 10,000 units/mL penicillin/streptomycin, and 250 mg/mL fungizone (Gibco^TM^, Invitrogen, Thermo Fisher Scientific, Waltham, MA, USA). The mouse pre-osteoblast cell lines MC3T3-E1subclone 4 (ATCC, Manassas, VA, USA) were cultivated in the α-MEM, alpha-minimum essential medium, containing 10% FBS, fetal bovine serum, 10,000 units/mL penicillin/streptomycin, and 250 mg/mL fungizone (Gibco^TM^, Invitrogen, Thermo Fisher Scientific, Waltham, MA, USA) and osteogenic medium, DMEM, FBS 10%, 50 µg/ mLascorbic acid, 10 mM β-glicerophosphate, 10 nM dexamethasone. The cells were cultivated in a humidified atmosphere containing 5% CO_2_ at 37 °C until confluence, and then subcultured or used for the experiments. 

#### 2.4.2. Indirect Cytotoxicity Test

Indirect cell cytotoxicity was evaluated with MC3T3-E1 osteoblast cells and L929 fibroblast cells. The cells were cultured in the extracted medium from each material group compared with cells in the standard culture medium. Each material was cut into circular shapes sized 10 mm in diameter and immersed into an SFM, serum-free cultured medium for 1 day. Then, the extracted media were retrieved. The osteoblast and fibroblast were separately seeded at 1 × 10^4^ cells/well and cultured in a 24-well culture plate in 10% serum-containing α-MEM and DMEM for 16 h to allow cell attachment on the culture plate. After that, each cell type was continuously cultured with SFM for 24 h and subsequently changed to an extracted medium, except for the control plate that was continuously cultured in the standard culture medium. A WST-1 assay (WST-1; Roche, Darmstadt, Germany) was applied to quantify the number of remaining viable cells according to mitochondrial dehydrogenase activity. The sample was transferred to a microplate reader (BiotrakTM II Amersham/Bioscience Piscataway, NJ, USA) for optical density (OD) at 440 nm, and the amounts of the cells were calculated (n = 5/group).

#### 2.4.3. Direct Biocompatibility Test

The ability of osteoblast and fibroblast cells to attach, grow, and differentiate on the material’s surface was evaluated on both surfaces of materials, with both depending on the morphology of the surface. The surface with large or more porosity with BCP accumulated was tested for osteoblast cells, while the smoother surface with small and fewer pores was tested for fibroblast cells. On the top surface of the SR shell, the BF and the CP membranes were tested with fibroblast cells, and the bottom surfaces were tested with osteoblast cells. In contrast, the top surface of the AD membrane was tested with osteoblast cells, while the bottom surface was tested with fibroblast cells due to the side-specific property of the material, as described in the previous study [10]. 

#### 2.4.4. Preparing the Cell-Sheath Structure

The materials were cut into a circular shape of 10 mm in diameter. Then, the non-testing surface of the materials sheath was fixed to a well plate with agarose gel and the testing surface was immersed in fresh culture medium, i.e., α-MEM or DMEM for osteoblast and fibroblast cells, respectively for 24 h. The osteoblast or fibroblast cells were seeded separately on the cell-sheath structure with an amount of 1×10^4^/well in each experiment, depending on the direct biocompatibility testing assay, and incubated at 37 °C under a humidified atmosphere containing 5% CO_2_.

#### 2.4.5. Cell Attachment and Morphologies

The SR shell, the semi-resorbable membranes (BF and AD membrane), and the CP membrane were cultured for 6 h in culture medium α-MEM for osteoblastic cells and DMEM for fibroblastic cells to allow cell attachment to occur on the material surface. Then, the membranes were rinsed with PBS and fixed in 2.5% glutaraldehyde (Sigma-Aldrich, St. Louis, MO, USA) in PBS for 2 h. All samples were dipped in ethanol series of 50–100% to dehydrate them and then coated with gold-palladium. Cell attachment and the morphologies of osteoblast and fibroblast cells were examined via SEM (Quanta400, FEI, Brno, South Moravian Region, Czech Republic).

#### 2.4.6. Cell Viability

Cell adhesion and distribution were fluorescence stained and visualized under a confocal laser scanning microscope (CLSM). After cell seeding on the material surface for 1, 3, and 7 days, the osteoblast and fibroblast cells were stained with fluorescein diacetate (FDA, Sigma-Aldrich, St. Louis, MO, USA). The materials were kept in darkness for 5 min and then cell washed twice with PBS and transferred to a glass slide. Cell viability, distribution, and proliferation were observed under a confocal laser scanning microscope.

#### 2.4.7. Cell Proliferation

The numbers of the vital cells on the cell-sheath structure of the material on days 1, 3, 7, 14, and 21 were determined using WST-1 assay. On the experiment day, the cell-sheath structure was rinsed with PBS and then incubated in 200 μL of the fresh culture medium with 20 μL of WST-1 solution for 4 h in 5% CO_2_ at 37 °C. From each well, 100 μL of solution was used to measure the absorbance of the formazan product using the microplate reader at 440 nm. The standard curve was used to calculate the numbers of cells from the levels of OD (n = 5/group/time point).

#### 2.4.8. Osteoblast Cell Differentiation

The osteoinductive properties of the fabricated materials on cell differentiation were evaluated. The osteoblast cells were seeded on fabricated materials and the CP membrane, and were cultured in an α-MEM. The cells on a plate cultured in the culture medium were the negative control, while those in the osteogenic medium were the positive control. The test was performed on culture-days 1, 3, 7, 14, and 21.

On the testing day, the cell-sheath structures were washed in PBS twice and then incubated in 200 µL of 1% Triton X-100 in PBS for cell lysis by the freezing and thawing method for three cycles of the minced cell-sheath structures. The cells were then lysed, and the mixtures were centrifuged at 2000× *g* for 10 min. The supernatants were kept at −80 °C for analyses of protein content, ALP activity, and osteocalcin.

- Total intracellular protein content

The cell lysis solution of each group was measured for total intracellular protein synthesis using the Bio-Rad Protein Assay kit (Bio-Rad, Grand Island, NY, USA). Quantification of total protein content (mg) was performed according to the manufacturer’s instructions, and the absorbance of solutions was read by using a microplate reader (BiotrakTM II Amersham/Bioscience Piscataway, NJ, USA) at 650 nm.

- Alkaline phosphatase activity (ALP assay)

ALP activity in cell lysis solution was detected by the Alkaline Phosphatase liquicolor (AMP Buffer, IFCC) Humazym Test (Human diagnostic worldwide, Wiesbaden, Germany) on culture days 1, 3, 7, 14, and 21. Then, 20 µL lysis solution was incubated in 1000 µL buffer solution (2-Amino-2-methyl-1-propanol, pH 10.4, magnesium acetate, zinc sulfate, and sodium azide) for 1 min at 37 °C. Next, 250 µL of substrate solution (p-Nitrophenyl phosphate, sodium azide) was added and mixed well. The optical density (OD) of these solutions was read after 1, 3, and 30 min and detected at 405 nm. The levels of ALP activity were standardized with the total protein amount and presented as mg p-nitrophenol/mg protein.

- Osteocalcin assay

The quantification of osteocalcin activity of the osteoblast cells was performed on culture days 1, 3, 7, 14, and 21 by Mouse Osteocalcin Enzyme Immunoassay kit (Alfa Aesar, BT-470, Tewksbury, MA, USA) following the manufacturer’s protocol. Results are reported as nanograms per milliliters (ng/) mL.

- Mineralization assay with Alizarin red staining

Calcium deposition was determined by alizarin red staining (Sigma-Aldrich, St. Louis, MO, USA) on culture-days 3, 7, 14, and 21. The cell-sheath structure was washed with 1× PBS, and then cells were fixed with 4% formaldehyde. After that, 1 mL of alizarin red solution (2 g in 100 mL of distilled water to adjust the pH to 4.1–4.3) was added to the cell sheath in a dark environment at room temperature for 20 min. The alizarin red was gently washed from 48 well plates with distilled water to wash out the red color. Next, the cell sheaths were observed under a fluorescence microscope (Nikon Eclipse Ti-S, Tokyo, Japan). The calcium deposition was quantified and calculated from the standard curve.

### 2.5. Statistical Analysis

The data were analyzed by using SPSS, version 23.0 software (IBM, Armonk, NY, USA) and presented as mean ± standard deviation. The differences in the parameters among the groups were tested by One-way Analysis of Variance (ANOVA) followed by Tukey HSD or DunettT3. The differences in the same group at different time frames were tested by a repeated measure of ANOVA and a paired T-test. Statistical significance was set at a *p* < 0.05.

## 3. Results

### 3.1. Material Morphology

**Figure 3:**
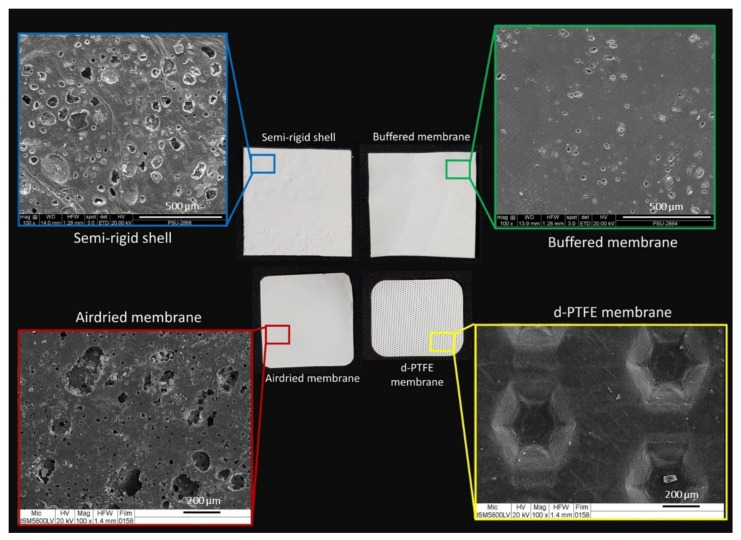
Gross observation of materials and SEM images at magnification ×100 (in the box area) of the SR shell (**upper left**), the BF membrane (**upper right**), the AD membrane (**lower left**), and CP membrane (**lower right**).

The surface of the fabricated material had different morphologies on the two sides. The SR shell and the BF membrane presented the rough bottom surface with BCP particle diffusion and a smooth top surface. The AD membrane had the opposite characteristics to the SR shell and the BF membrane, it had a smooth bottom surface and a rough top surface. In contrast, the CP membrane was smoother on both surfaces than the fabricated material and had dimples on the top surface for enhancing soft tissue attachment. The appearances of the materials are presented in Figure 3.

SEM images of the CP membrane manifested a smooth texture, while those of the fabricated shell and membranes exhibited tiny pores and diffusion of BCP particles on the surfaces. The SR shell presented a rougher surface than the BF and the AD membranes. The AD membrane had more and larger porosity than the BF membrane, as shown in Figure 3.

### 3.2. Indirect Cell Cytotoxicity

**Figure 4:**
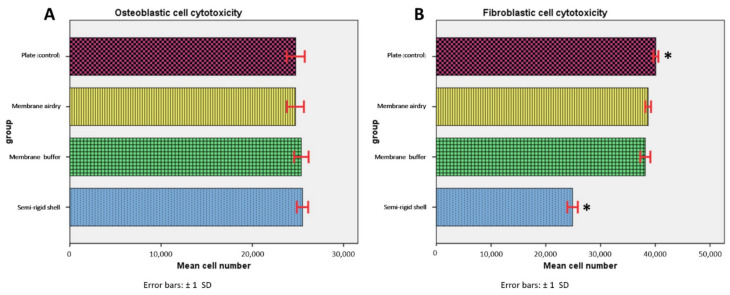
Indirect cell cytotoxicity of the osteoblast (**A**) and the fibroblast cells (**B**) of the SR shell, the BF membrane, the AD membrane, and a culture media. * Significant difference among groups at *p*-value < 0.05.

After osteoblast and fibroblast cells had been cultured for 24 h in extracted medium, all groups presented comparable numbers of osteoblast cells without statistically significant differences (*p* > 0.05) among the groups. For fibroblast cells, the SR shell had significantly fewer fibroblast cells than the control (*p* = 0.000), although the cell number was still not less than 50% of the total amount, which indicated no cytotoxicity of the material. The results are shown in Figure 4.

### 3.3. Cell Attachment and Morphology

**Figure 5:**
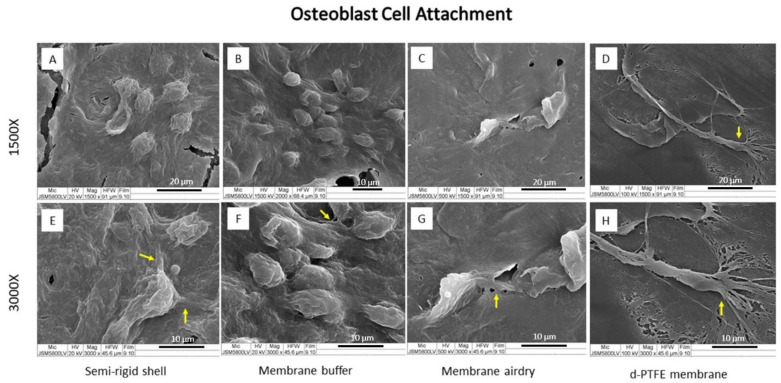
SEM images of osteoblast cell attachment on the SR shell (**A**,**E**), the BF membrane (**B**,**F**), the AD membrane (**C**,**G**), and the CP membrane (**D**,**H**) at magnification ×1500 (**A**–**D**) and ×3000 (**E**–**H**). The yellow arrows indicate the spreading of filopodia.

**Figure 6:**
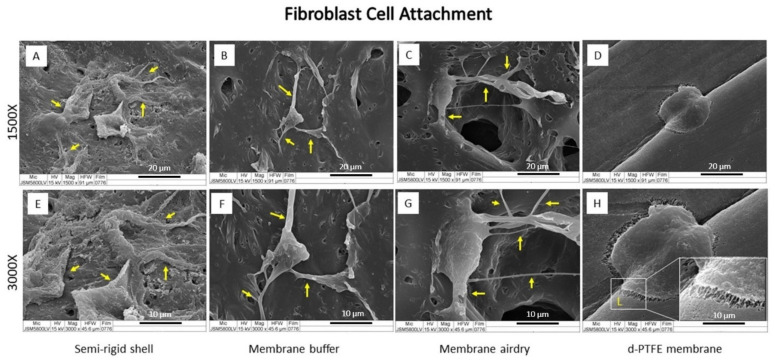
SEM images of fibroblast cell attachment on the SR shell (**A**,**E**), the BF membrane (**B**,**F**), the AD membrane (**C**,**G**), and the CP membrane (**D**,**H**) at magnification ×1500 (**A**–**D**) and ×3000 (**E**–**H**). The yellow arrows indicate the filopodia spreading, and the lamellipodia in the white box (**H**).

After cell seeding and culture for 6 h, the SEM images showed good cell attachment of the MC3T3-E1 osteoblast as well as L929 fibroblast cells on the material surface, as shown in Figure 5 and Figure 6, respectively.

In osteoblast cell culture (Figure 5), the CP membrane presented the extension of osteoblast filopodia more than the fabricated materials. All fabricated materials presented a rounder shape osteoblast cell but had more cell accumulation than the CP membrane. Fibroblast cells attached well to the surface of all materials. The SR shell demonstrated a spindle-like shape with filopodia expansion more than the BF and AD membranes, while the CP membrane showed a flat round shape with the spreading of lamellipodia (Figure 6).

The morphology and the number of osteoblast and fibroblast cells on the material surface after culturing for 6 h indicated that both osteoblasts and fibroblasts were well attached to fabricated materials, as well as to the CP membrane.

### 3.4. Cell Viability

**Figure 7:**
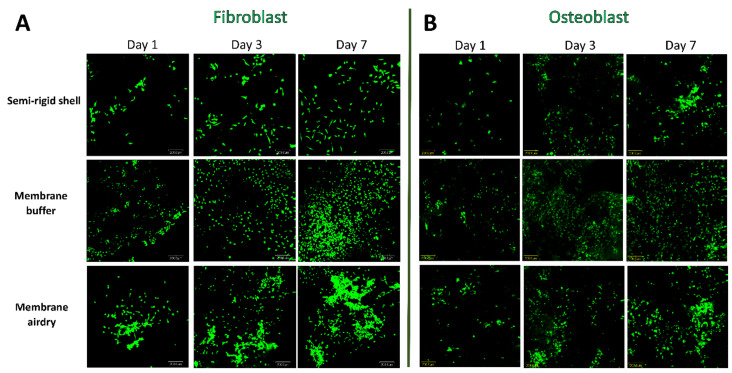
Fluorescence images of fibroblast (**A**) and osteoblast (**B**) cell viability in the SR shell (**upper rows**), the BF membrane (**middle rows**), and the AD membrane (**lower rows**) on day1, day3, and day7.

Cell viability on each fabricated material was observed under a confocal laser scanning microscope with fluorescence staining. The fibroblast and osteoblast cell viability of the fabricated shell and membranes is presented in Figure 7. Live osteoblast and fibroblast cells present green spots with FDA staining. On day 1 of both osteoblast and fibroblast live cells were noticed from the presentation of green spots on the surfaces of the fabricated materials. On days 3 and 7, increasing amounts of green areas were observed. However, the AD and the BF membranes seemed to have greater numbers of cells than the SR shell. These results demonstrate the biocompatibility of the fabricated materials, which was confirmed by the growing attachment and proliferation of the cells on the surface of the fabricated material.

### 3.5. Cells Proliferation

**Figure 8:**
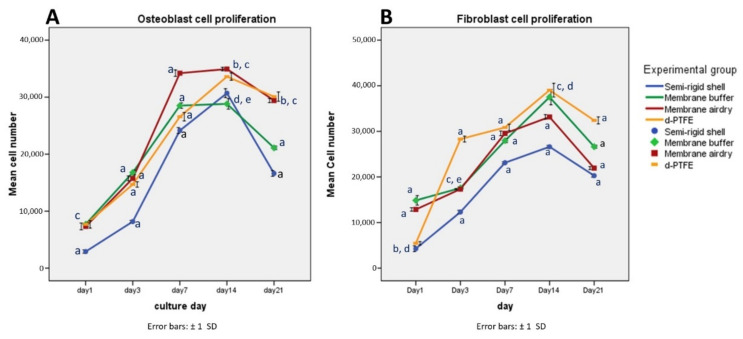
Osteoblast (**A**) and fibroblast (**B**) cell proliferation of the SR shell, the BF membrane, the AD membrane, and the CP membrane on days 1, 3, 7, 14, and 21. *p* < 0.05 was set as statistical significance. a = significant difference from other groups, b = significant difference from the BF membrane, c = significant difference from the SR shell, d = significant difference from the AD membrane, e = significant difference from the CP membrane.

Osteoblast cell proliferation is shown in Figure 8. The results presented the same proliferation pattern in all material groups. The number of cells increased from day 1, reaching the highest numbers on day 14 before decreasing by day 21; this was possibly due to some cell death from tight cell growth on the material surface. On day 21, the CP membrane presented significantly greater cell numbers than the BF membrane and the SR shell (*p* < 0.05) but were not statistically different (*p* > 0.05) from the AD membrane. The SR shell showed significantly fewer osteoblast cells than the other groups at all time points (*p* < 0.05).

Fibroblast cell proliferation is shown in Figure 8. All fabricated material groups presented similar fibroblast cell proliferation patterns. The number of cells increased from day 1, reaching a peak on day 14 and then decreasing by day 21. The CP membrane presented significantly greater cell numbers than the BF membrane, the AD membrane, and the SR shell (*p* < 0.05). The SR shell showed significantly fewer fibroblast cells than other groups at all time points (*p* < 0.05).

### 3.6. Osteoblast Cell Differentiation

**Figure 9:**
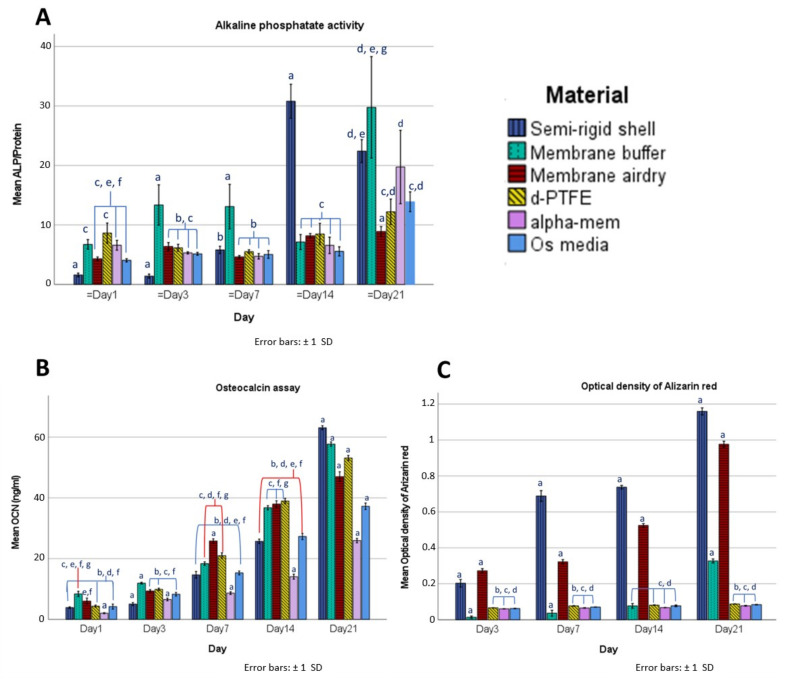
Alkaline phosphatase activity (**A**), Osteocalcin assay (**B**), and Alizarin red staining (**C**) of the SR shell, the BF membrane, the AD membrane, the CP membrane, the α-MEM (a negative control group), and the osteogenic media (a positive control group). Statistical significance was set at *p* < 0.05. a = significant difference from other groups, b = significant difference from the BF membrane, c = significant difference from the SR shell, d = significant difference from the AD membrane, e = significant difference from the CP membrane, f = significant difference from the α-MEM, g = significant difference from the osteogenic media.

Osteoblast cell differentiation was evaluated according to alkaline phosphatase (ALP) activity, osteocalcin assay, and alizarin red staining. The results are shown in Figure 9.

ALP is a by-product of osteoblast cell metabolism; an increasing level of ALP activity can be considered representative of active bone formation activity, and is a marker for early osteoblast cell differentiation. All groups showed a trend of increasing ALP activity over time. The SR shell group reached the highest level of ALP on day 14, before dropping on day 21, with possibly too much confluence of cells leading to cell death. Meanwhile, the BF membrane had a higher level of ALP on day 21 than the SR shell, the α-MEM, the osteogenic medium, the CP membrane, or the AD membrane.

Osteocalcin (OCN) is a noncollagenous protein and a marker for the late differentiation of osteoblast cells. The level of osteocalcin increased continuously in all groups from day 1 to day 21. The SR shell presented the highest osteocalcin secretion, followed by the BF membrane, the CP membrane, and the AD membrane. All of the material groups showed significantly higher osteocalcin levels (*p* < 0.05) than the α-MEM (the negative control) or the osteogenic media (the positive control).

The mineral secretion of the osteoblasts was visualized and quantified by alizarin red staining. The calcium content increased in all groups from day 3 to day 21. The SR shell presented the highest calcium content, followed by the AD, BF, CP membranes, and the control groups. The fabricated materials presented significantly higher calcium content than the α-MEM or the osteogenic media (*p* < 0.05), but the CP membrane showed no significant difference (*p* > 0.05) from the control groups.

The SR shell and the BF membrane had better osteoblastic cell differentiation than the commercial CP membrane, the α-MEM (a negative control), or the osteogenic medium (a positive control).

## 4. Discussion

A new concept for a semi-rigid shell barrier system was introduced, and the materials were tested. The system comprises a semi-rigid shell fixed to the outer cortex of the defect, which serves as a new bone wall and contains the graft particles. Subsequently, the structure is covered by covering membranes, i.e., the buffered membrane, or the airdried membrane for excluding unwanted cells. The three structures of the barrier, i.e., an SR shell, a BF membrane, and an AD membrane, served to further test the biocompatibility in vitro to support the hypothesis that the membrane was bioactive. Appropriate physicochemical characteristics and mechanical strength were observed, as presented in a previous study [10].

### 4.1. Material Morphology

The fabricated materials had diverse surface topographies on their two sides. The surface architectures and pore structures of the materials were designed to support different applications. The smooth surface was suitable for connective tissue and functions as a barrier, while rough surfaces were aimed to support bone regeneration, and porosity supported angiogenesis. The surface roughness and the diffusion of BCP particles facilitated cell attachment, and consequently promoted bone formation [20].

### 4.2. In Vitro Biocompatibility and Cell Cytotoxicity

All fabricated materials demonstrated a lack of cytotoxicity to both osteoblast and fibroblast cells, in accordance with the ISO 10993-5 (year 2009) standard, which states that the cell lysis of the testing materials in the extracted medium after 24 h of incubation must be less than 50% [21,22,23].

The feature of multiple filopodia of osteoblast and fibroblast cells (as shown in Figure 5 and Figure 6, respectively) indicated that the surface topography of the materials favored cell attachment and function. Factors that influence cell attachment and proliferation include surface roughness, microscale, nanoscale topography, and surface energy [24].

Interactions among cells and the surface materials involve protein adsorption on the surface, the initial contact between cells and the adsorbed protein layer, RGD-integrin binding, and slight cell spread [25,26]. After cell attachment, the cells initially spread and migrated by circumferential extension of actin filaments, called lamellipodia. In this stage, cells typically formed broad and flat shapes, and protruded finger-like appendages of the cytoskeleton called filopodia. Filopodia is the cell signaling sensor that is used to receive and send the cell substrate to regulate cell function [27].

### 4.3. Cell Viability, Proliferation, and Osteoblast Cell Differentiation

Cell viability is a measurement of the number of living cells in a population [28,29]. Fluorescent staining is a qualitative tool for visualizing cell viability. A dye can be visible in the condensing and spreading area of living cells via the mechanism of intracellular esterase hydrolysis [23]. Meanwhile, the WST-1 assay that evaluated the mitochondria mechanism serves to quantify cell viability. The proportion of the dark color of formazan crystals indicates the number of viable cells which represents cell proliferation [30].

All fabricated materials demonstrated an increase in the fluorescence staining of osteoblast and fibroblast cells from day 1 to day 7, indicating that the material surface favored cell attachment and proliferation. The cell proliferation assay in all material groups showed an increased number of osteoblast and fibroblast cells with time, indicating the cytocompatibility of the semi-rigid shell barrier system.

In this study, the fabricated SR shell, the AD, and the BF semi-resorbable covering membranes had rough surfaces with a micro-architecture that favored osteoblast cells, while the smooth surface of the commercial d-PTFE membrane was more favorable to fibroblast cells. This supported the hypothesis that osteoblast cells on rough surfaces exhibit more osteoblastic differentiation characteristics compared to smooth surfaces, such as greater ALP activity and OCN [31].

The fabricated materials presented high ALP activity, OCN, and calcium secretion of alizarin red staining, particularly the semi-rigid shell, that supported osteoblastic differentiation and function. The fabricated materials composed of polymer and BCP components induced a greater osteoblastic differentiation function than commercial d-PTFE, which is a pure polymer. The increase of MC3T3-E1 osteoblast cell chemotaxis occurred when there was a secretion of calcium concentration in the extracellular matrix in the range of 1.8–5 mM [32]. Moreover, an inorganic phosphate signaling molecule upregulated osteoblast cell differentiation by increasing the phosphate ion concentration [33], which increased the collagen and calcium content in the culture media [34]. However, there is no optimal concentration of calcium and phosphate ions for successful osteogenesis [35]. The surface roughness of the fabricated materials enhanced cell adhesion and favored cell proliferation, expression of the osteoblastic differentiation phenotype of higher ALP activity, and OCN level, compared to the smooth architecture of the CP membrane. Hence, the semi-rigid shell barrier system is biocompatible, promotes proliferation and differentiation of osteoblast cells, and supports application in bone augmentation procedures.

The BF and the CP membranes exhibited similar patterns of fibroblast cell proliferation and osteocalcin production and presented less alizarin red than the SR shell and the AD membrane. The BF membrane was more likely polymeric than the SR shell and the AD membrane that contain numerous BCP components [10] and possess osteoinductive characteristics. Some constructed barrier membranes, such as PLA and PLGA, are resorbable membranes. In 2020, Zimina [36] reported that pure PLA polymeric membranes favored mesenchymal stromal cell (MSC) proliferation, i.e., via the same pattern as BF and CP. Meanwhile, a PLA/HA membrane that had calcium phosphate favored osteoblasts, which agreed with the AD and SR shell. The results also agreed with research by Yang [37] who, in 2009, reported that nano-apatite (nAp) incorporated with a PCL membrane significantly enhanced bioactivity and promoted osteoblast-like cell proliferation and differentiation more than pure PCL membranes. Moreover, the fabricated materials also presented good cytocompatibility, i.e., osteoblast and fibroblast cell proliferation increased from day 1 to day 14. This result was better than that obtained by Rothamel et al., who found that PDL fibroblast cells and human osteoblast-like cell proliferation on different commercial collagen membranes decreased after day 7 [38]. Some commercial membranes are made of polymers such as poly-D, L-lactide-co-glycolide (Resolut adapt^®^), poly D-L, L polylactic acid (Epi-Guide^®^) and poly-D, L-lactide/poly-L-lactide blended with acetyl tri-n-butyl citrate (Guidor^®^). Only PTFE, a polymer membrane, is a non-resorbable membrane. However, no commercial membrane on the market is a semi-resorbable and an osteoinductive membrane. Those resorbable membranes have drawbacks such as stiffness, distortion, and the creation of an acidic environment, making them unpopular [12]. Meanwhile, collagen membranes become popular due to their compatibility with soft tissue but have drawback of easily collapse [39]. PTFE is well accepted, but still has to be removed and is prone to exposure [40]. Therefore, the search for an ideal membrane is ongoing.

In summary, we provided evidence that the semi-rigid shell barrier system containing BCP particles has osteoinductive properties that induce osteogenic cell differentiation and consequently promote bone formation in vitro.

## 5. Conclusions

A semi-rigid shell barrier system based on polycaprolactone and biphasic calcium phosphate demonstrated good in vitro biocompatibility, no cell cytotoxicity, good cell attachment, and proliferation. It also demonstrated better osteoblast cell differentiation than d-PTFE. Further study in an animal model and in clinical situations would confirm the benefits of the proposed system. 

## Figures and Tables

**Table 1:** Experimental groups.

Study Groups	Abbreviation
Semi-rigid shell	SR shell
Semi-resorbable barrier membrane fabricated using a buffering technique	BF membrane
Semi-resorbable barrier membrane fabricated using an air-dry technique	AD membrane
Commercial d-PTFE membrane (Cytoplast^®^, USA)	CP membrane

## Data Availability

Not applicable.
